# Impact of hydrokinetic turbines on rainbow trout behaviour

**DOI:** 10.1038/s41598-026-43568-8

**Published:** 2026-03-16

**Authors:** Guglielmo Sonnino Sorisio, Stephanie Müller, Catherine A. M. E. Wilson, Jo Cable, Pablo Ouro

**Affiliations:** 1 School of Engineering and Water Research Institute, Cardiff University, Queen’s Buildings, 14-17 The Parade, Cardiff, CF24 3AA UK; 2 School of Biosciences and Water Research Institute, Cardiff University, Cardiff, CF10 3AX UK; 3 School of Engineering, University of Manchester, Manchester, M13 9PL UK

**Keywords:** Civil engineering, Environmental impact, Animal migration, Behavioural ecology, Freshwater ecology, Hydroelectricity

## Abstract

Hydrokinetic turbines are an emerging technology to supply reliable and renewable energy by harnessing kinetic energy from river or tidal flows. They are a potential alternative to current hydropower schemes that impound rivers, cause habitat fragmentation and fish mortality. To-date there is limited knowledge available about fish behaviour in the vicinity of hydrokinetic turbines and their effects on shoaling. In this study, juvenile rainbow trout (*Oncorhynchus mykiss*) were allowed to swim either individually or as a shoal of three fish in a laboratory flume with five turbine configurations of single and paired turbines. Results show the turbines did not reduce fish passage when in operation, and blade strikes were rare, none causing significant harm. Fish were more likely to swim in the turbine’s downstream wake and upstream of the turbine (known as bow wake) when two turbines were operating because of the generation of larger areas of low velocity. Fish approached the turbines more often in shoals than alone and were overall bolder in shoals. This study suggests that hydrokinetic turbines do not significantly affect fish movement, thus supporting their utilisation as a suitable technology to extract renewable energy from rivers.

**Supplementary Information:**

The online version contains supplementary material available at 10.1038/s41598-026-43568-8.

## Introduction

Reliable power supply is essential to modern society, and affordable and clean energy is one of the United Nations Sustainable Development Goals (SDGs)^1^. Most large cities and settlements have access to national energy grid networks and power plants that deliver a stable power supply to homes and industry through a mixture of fossil, nuclear, and renewable energy. Remote communities, however, still struggle with reliable power supply and may depend on renewable resources that may not be predictable. The SDGs aim for a reduction in fossil fuel usage^1^, favouring an increase in renewable and low carbon resources, ideally using resources that do not damage the natural environment.

Freshwater watercourses are a valuable resource of energy which has a long history of being exploited by humans. The most common form of energy extraction from rivers in modern times is by impounding large volumes of water with a dam and forcing it through turbines to generate power. Such hydropower plants generate 14.3% of the world’s electricity, but their contribution varies widely by country and region, ranging from 0% to 100%^2,3^. Hydropower plants are considered a low carbon energy source but they still have a negative effect on the rivers, causing fragmentation, preventing and/or delaying fish migration, disrupting natural sediment transport characteristics, and causing fish mortality^4,5^. Over 3,000 new hydropower plants are planned^6^, particularly in relatively untapped rivers such as the Amazon, Congo, and Mekong^7^, home to a third of freshwater species, some found nowhere else. Hydropower plants installations without any technical or natural fish passage solution, however, can cause up to 100% mortality in freshwater fish migrating through turbines^8^ and block the migration of others^5^, with small dams having the largest ecological impact relative to capacity^6,9^.

For remote communities, not connected to the wider grid and in need of a reliable source of power, hydrokinetic turbines are emerging as an alternative and less environmentally-damaging alternative to hydropower plants^10^. Hydrokinetic turbines use the flow velocity of rivers to generate power. They do not impound rivers or form a barrier that spans the full river width^11^. Hydrokinetic turbines are also easier and cheaper to install than hydropower plants with potentially fewer environmental drawbacks and predictable power delivery^11,12^, making them suitable for use in remote regions^9,12^. Several small hydrokinetic turbines exist and currently operate in countries like Brazil and Malaysia^10,13–15^. These are used to provide power to small communities and are effective in shallow but fast flowing streams. The designs are of various types but mainly consist of horizontal and vertical axis turbines and sizes range from diameters of 0.36 to 4.4 m^13–18^.

Although hydrokinetic turbines potentially have less impact on river hydrodynamics and geomorphology^19^, they still block part of the watercourse, and therefore we must seek to understand how they alter the flow field and associated habitats before this technology is widely adopted. Vertical Axis Turbines (henceforth referred to as VATs) are a typology of hydrokinetic turbines^20,21^ that can generate energy from relatively low flow velocities of 1.5 m/s or lower^22^. VATs have little effect on the flow when stationary, making them suitable for a wide range of rivers while their horizontal axis counterparts operate at higher rotational speeds and require velocities above 2.0 m/s. The effects of full-scale operating turbines on the flow have been studied, e.g. with a turbine deployed in Alaska which created an induction zone upstream where the upstream velocity decreased while turbulence increased compared to the surrounding flow^23^. Downstream of this turbine, the near wake had a fast recovery, quickly turning into a low energy far wake^23^.

Numerical and experimental studies of VATs have characterised the wake behind a varied range of turbine configurations; a single turbine featured a small stagnation point upstream of the turbine^24–26^ with a downstream wake that expanded laterally bounded by regions of high vorticity, which started to converge at 2.5 turbine diameters (2.5D) downstream with vortices reaching a maximum size of 0.85D^24^. A VAT wake can be categorised into three main regions: the near wake extending 2D from the turbine featuring low momentum and bounded by high energy vortices, a transition region between 2D-5D downstream of the turbine where there is momentum recovery but still maintains a high level of turbulence and where the wake expands vertically, and the far wake beyond 5D where the rotor-averaged velocity is recovered to 90–95% with some minor fluctuations^21^. Using twin VATs (TVAT) can generate a synergistic effect that potentially generates more energy compared to a single turbine. The interactions between the wakes of the two turbines depends on their relative rotational direction. When rotating in the same direction (co-rotating), there is a reduced velocity in the wake compared to a single turbine and there is the largest lateral expansion of the wake, whereas counter-rotating turbines will have the fastest wake velocities and momentum recovery by 5D if counter-rotating forwards or a wake with a large vertical extension if counter-rotating backwards^27^.

The limited knowledge offish behaviour around hydrokinetic turbines is based on laboratory flume, river, and tidal turbine studies. In flume trials and rivers, salmon and rainbow trout avoided the turbine when operational, but passage remained unaffected^20,29–33^. In tidal channels, a 35% decrease in turbine interactions was observed when the turbine was operational^34^. Other field studies, however, reported attraction to the turbines and increased presence of fish in the wake^34,35^ and a 378% increase in shoaling behaviour^35^. Such aggregations can create predation hotspots^36^. Mostly, fish prefer to pass to the side^31,37,38^ or above the turbine when spatially constrained^39^. In a narrow flume^31^ or in a tidal channel^40^, turbine presence did produce a movement barrier for rainbow trout. Survival rates for turbine exposure are generally reported as being above 95%^29–31,33,37,41^ but blade strike risks can vary widely in laboratory and numerical studies from 0–1.3%^38,42^ to 6–68.9%^41,43^. Injury rates from hydrokinetic turbines also vary, with maximum values reaching 20% with bruising and descaling being the most common form of injury^30^. While the hydrokinetic turbine sound does not appear to impact fish responses, visual cues and turbine visibility do affect behaviour^34,37,42^.

Few studies have analysed the effects of fish shoaling near a hydrokinetic turbine^34,35^, even though most fish species are shoaling. Hence, this effect should be considered when evaluating fish behavioural responses to the presence of hydrokinetic turbines. The primary drivers of fish shoaling are well understood to be predation avoidance^44–48^, foraging^47^, social interactions^45^, and hydrodynamic benefits^49,50^. However, the interplay between these factors is debatable^45,46,49^ and this balance will shift according to context. With regard to hydrokinetic turbines and moving flow, shoaling might bring a primarily hydrodynamic benefit^49,50^. Fish can reduce their energy expenditure by up to 53% in shoaling Giant danio (*Devario aequipinnatus*)^51^. Experimental studies^52–55^, a fish and a flapping foil^56,57^, and numerical studies^58,59^ have also found energetic benefits of shoaling, but the driving mechanism of the energy reduction of shoaling is unclear. The leading fish in a shoal can also benefit from shoaling due to the reduced pressure difference generated by the fish behind^58^. A more common explanation for the hydrodynamic benefits, however, is that following fish can exploit the lower drag force and the reverse Karman street vortices in the wake of the leading fish. They achieve this by swimming directly behind the fish in front^56,60^ or in a staggered or diamond formation^49,59^. Fish in a shoal could occupy any position and gain energetic benefits. As positioning is dynamic, this has led to arguments against fish shoals ‘drafting’ in the vortex street^53,61^. There are studies, however, that have not found any metabolic difference caused by shoaling in rainbow trout^52^, demonstrating that the principles discussed may not be universally transferable among species and flow conditions. Other factors affecting shoaling are the familiarity and geographic origin of the fish with its shoal mates^62,63^, infection status^48,64,65^, and presence of predators^48^.

There is still a knowledge gap concerning hydrokinetic turbine configuration and shoaling in the context of hydrokinetic turbines that needs to be filled before these devices are implemented in sensitive ecosystems. This study examines the behaviour of juvenile rainbow trout (*Oncorhynchus mykiss*) in the presence of five different configurations of single or twin laboratory scale VATs, and compares scenarios for a single fish and for a shoal of three fish. This species’ native range covers North American countries and their introduced range includes almost all other continents where this technology may be useful, moreover rainbow trout are an established model species for fish behaviour and shoaling^44,48,52,62^ and are closely related to migratory salmonids like steelhead (*Oncorhynchus mykiss*). The aim is to provide a new understanding of how twin-turbines and their combined hydrodynamics affect fish behaviour alone or while shoaling, and whether shoaling affects fish ability to navigate complex flow fields generated by laboratory scale turbines.

## Results

### Distance, exploration and passage

**Fig. 1:**
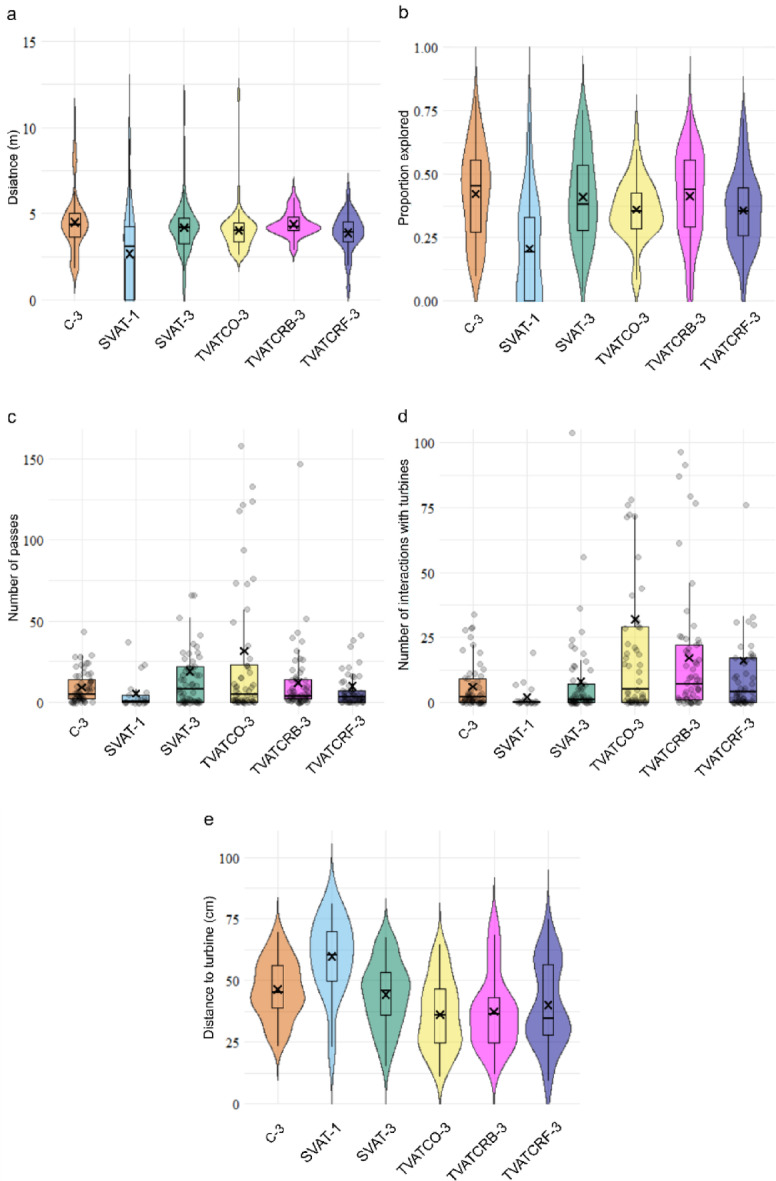
Violin and Boxplots describing, for each treatment: **(a)** Distance swam by fish; **(b)** The proportion of the working area explored by fish; **(c)** The number of times each fish passes from downstream to upstream or from upstream to downstream of the turbines; **(d)** Number of times fish entered the turbine area; **(e)** The average distance between fish and the nearest turbine throughout the trial. In all cases, the box and violin plots show median, interquartile range, and 95th percentile and the cross marks the mean.

**Fig. 2:**
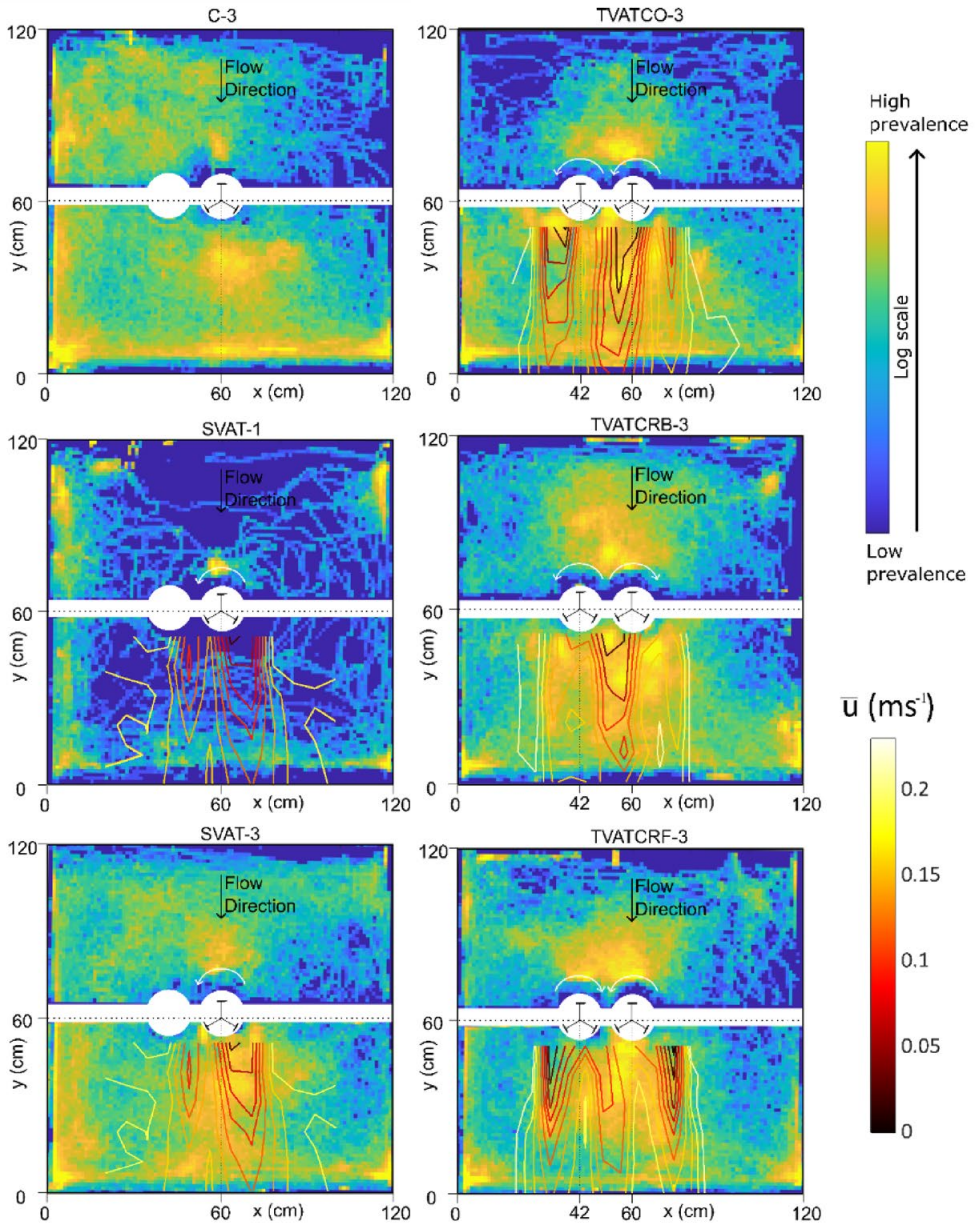
Heatmaps showing the prevalence in spatial occupation of the experimental area by the fish and the turbine wake overlaid to show the streamwise velocity borders downstream of the turbines. Colour scales for both the density plot and the streamwise velocity are given to the right of the plot. For each treatment, data from all fish is combined, thus treatments with single fish have less data. The white arrows indicate turbine rotational direction.

Fish swam the largest distance in the control conditions (C-3, non-rotating turbine with fish shoal) with a mean of 4.5 m (C-3, Fig. 1a), and significantly less in treatments SVAT-1 (single turbine and single fish), TVATCO-3 (co-rotating twin turbines with fish shoal), and TVATCRF-3 (counter-rotating twin turbines with fish shoal) within the working section of the flume in comparison with C-3 (GLMM, *p* < 0.02). This is reflected in the proportion of the total working area explored by each fish (Fig. 1b), where the fish in C-3 on average explored significantly more of the working area (average of 40%) than the fish in SVAT-1 (20% on average), and TVATCO-3 and TVATCRF-3 (26% on average), which was significantly lower than all other treatments too (GLMM, *p* < 0.017). The decreased distance and exploration did not, however, affect passage success past the turbines (Fig. 1c), all treatments statistically having the same passage as control C-3, except for TVATCO-3 that had significantly more (GLMM, *p* = 0.001). Figure 1d shows fish entering the turbine area (52 < *x* > 68 cm, 52 < *y* > 66 cm in Fig. 5) compared to C-3, with SVAT-1 having significantly fewer interactions with the turbine (GLMM, *p* = 0.002) whereas SVAT-3 had more than C-3 (GLMM, *p* = 5e-6), TVATCRB-3 and TVATCRF-3 had significantly more than C-3, SVAT-1 and SVAT-3 (GLMM, *p* = 2e-16). Notably, TVATCO-3 had the most fish entering the turbine area out of all treatments (GLMM, *p* = 2e-26). This finding correlates with the amount of time spent very close to the turbines (blue rectangle in Fig. 5), with fish in TVATCO-3 and TVATCRF-3 spending significantly more time there (GLMM, *p* < 0.03). Figure 1e shows the same trend in the results from the relationship between treatment and distance to the turbines, where fish in SVAT-1 stayed significantly further away from the turbine than all other treatments (Fig. 2; GLMM, *p* < 0.005), and the three treatments with twin turbines staying significantly closer than control (GLMM, *p* < 0.008).

### Time in the working area

In all treatments, fish spent approximately 25% of time upstream and 75% downstream of the turbines (GLMM, *p* > 0.12). Part of the downstream time was spent resting on the flow straightener, where fish in SVAT-1 spent significantly more time resting (GLMM, *p* = 0.007) and fish in TVATCRB-3 spent significantly less time resting (GLMM, *p* = 0.003) compared to the control (C-3). Time spent in the near wake was highest in the three treatments with twin turbines (GLMM, *p* < 0.005) with a mean value of 123 s which is 20.5% of the experiment’s duration, while the rest of the shoaling treatments were statistically the same as the control (Fig. 2). Time in the far wake, however, did not vary across treatments, except for TVATCRF-3 where it was 45.8% lower than control (GLMM, *p* = 0.044). Time in the bow wake (Fig. 2) was the statistically the same for all treatments except for TVATCO-3 in which the fish spent more time there, 20 s on average (GLMM, *p* = 0.0015). Areas of low velocity in the wake of the turbines shown in Fig. 2 is generally associated with high prevalence, this can be seen to extend to the flume’s lateral walls, indicating that the fish were seeking low velocity areas.

### Tailbeat frequency

**Fig. 3:**
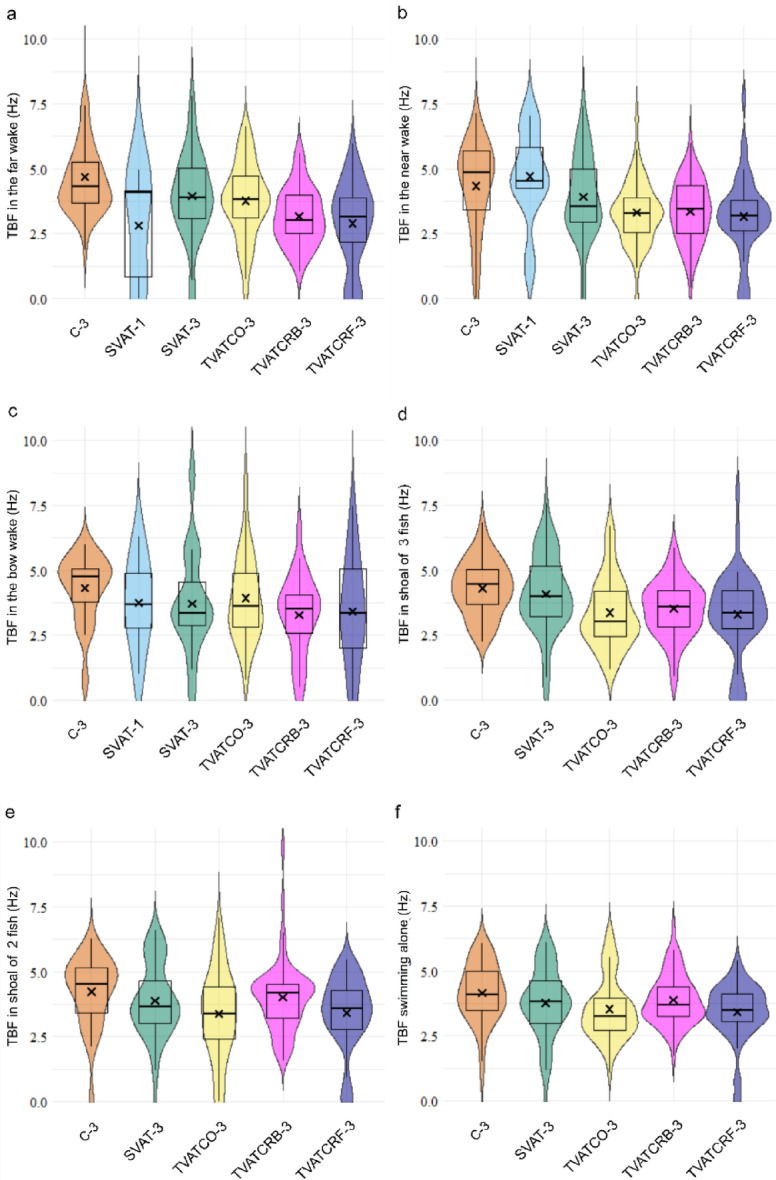
Results of average tailbeat frequency (TBF) for each fish for several swimming areas and shoal configurations for each treatment. **(a)** Far wake (yellow box in Fig. 5). **(b)** Near wake (green box in Fig. 5). **(c)** Bow wake (red box in Fig. 5). **(d)** When swimming in a shoal of three fish. **(e)** For the fish swimming in a shoal of 2 fish (disregarding the fish swimming alone). **(f)** When swimming alone. Boxplots show median, interquartile range, and 95th percentile and the cross marks the mean.

Overall tailbeat frequency (TBF) was 14.6% higher in C-3 than SVAT-1, SVAT-3, TVATCO-3 and TVATCRF-3 (GLMM, *p* < 0.02) throughout all swimming areas. In the far wake (Fig. 3a), a lower TBF than control with a mean of 4.9 Hz is present for treatments with rotating turbines (GLMM, *p* < 0.012). In the near wake, TBF was significantly lower than control only in treatments with twin turbines as these generated the largest low-velocity region (Fig. 3b; GLMM, *p* < 0.002). In the bow wake, there was no difference among any treatment in TBF with mean values ranging between 3.0 Hz and 4.5 Hz (Fig. 3c; GLMM, *p* > 0.08). When in a shoal of three fish (Fig. 3d), the combined TBF values for all shoaling fish in all three treatments with twin turbines were lower on average by 21% (GLMM, *p* < 0.005) than the TBF of 4.1 Hz found in the control (C-3) and single turbine cases (SVAT-1 and SVAT-3). When shoaling as a pair (Fig. 3e), fish swimming together had a lower TBF only in TVATCO-3 and TVATCRF-3, all other treatments being the same as control. When swimming alone, however, all treatments except TVATCRB-3 had a lower TBF than the control (GLMM, *p* < 0.02), as shown in Fig. 3f.

### Shoaling behaviour

**Fig. 4:**
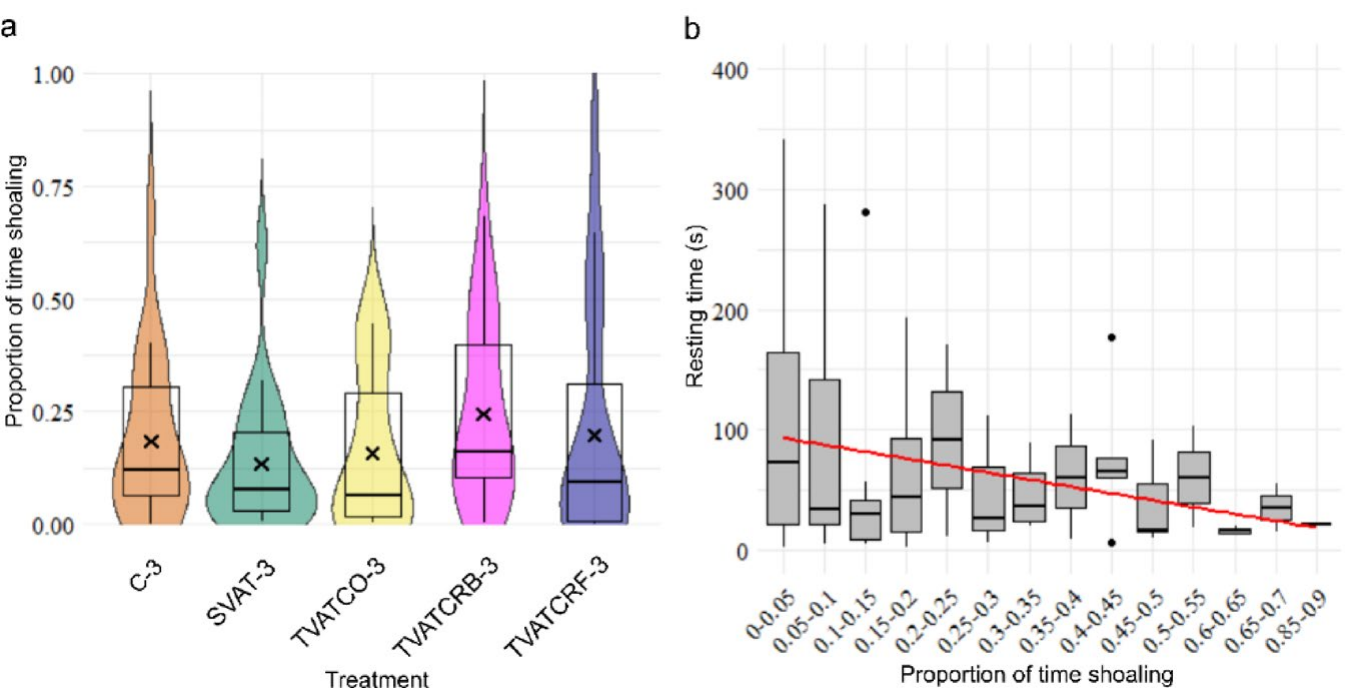
**(a)** The proportion of time spent shoaling (combined data for a shoal of three and a shoal of two) per treatment with treatment SVAT-1 excluded. **(b)** Negative correlation between the time spent resting on the downstream flow straightener and the proportion of time spent shoaling.

While shoaling, the proportion of time that an individual led was split equally between the three fish the longer they shoaled, with an individual only appearing dominant if the shoaling duration was short (GLMM, *p* = 0.005) and there was no difference in dominance of an individual between treatments (Fig. 4a; GLMM, *p* > 0.11). Shoaling, however, was associated with a reduced TBF in the bow wake (GLMM, *p* = 0.007) but not in the near and far wake (GLMM, *p* > 0.62). Shoaling fish also spent less time resting, (Fig. 4b; GLMM, *p* = 0.004), and were more likely to interact with the turbine (GLMM, *p* = 2.4e-14).

### Individual and shoal behaviour comparison

Individual fish swam significantly shorter distances than shoaling fish for the same single turbine configuration (Fig. 1a; GLMM, *p* = 0.02) and explored less of the working area (GLMM, *p* = 5.6e-6) but this did not translate to a reduced passage rate. Resting time (shown in Fig. 4b) was increased by an average of 181 s in SVAT-1 compared to SVAT-3 (GLMM, *p* = 0.003), with very few turbine interactions (GLMM, *p* = 0.002) and less time spent near the turbine (GLMM, *p* < 0.04). Despite the presence of shoalmates in SVAT-3, fish in SVAT-1 had the same TBF values in all areas (GLMM, *p* > 0.12).

## Discussion

For all tested configurations, the hydrokinetic turbines did not limit fish passage in a flume where the turbines blocked 25% of the cross-section. This is consistent with findings from laboratory and field studies evaluating hydrokinetic turbine passage^20,30–33^. Narrow channels, however, can constrict movement^31,40^ but in the relatively wide channel where neither turbine nor their wakes span across the entire cross-section, fish were able to use the wide undisturbed corridors without turbine influence to pass up and downstream. The passage data is further supported by the lack of differences in time spent upstream and downstream of the turbines across treatments. This is despite the decrease in distance covered and proportion of the area explored caused by presence of rotating rather than stationary turbines, but this may be explained by fish choosing to spend more time holding station in the turbine wake. The treatments for which distance covered was lower than the control (single fish, and shoals with co-rotating turbines and forward counter-rotating turbines) were the same in which either the amount of time spent resting was higher (SVAT-1) or the amount of time entraining in the near- and bow-wake was higher (TVATCO-3 and TVATCRF-3). TVATCO-3 was also the only treatment to have significantly higher passage rates than C-3 but still had lower distance and exploration, this may be explained by the increased time spent in the bow wake in which fish may have crossed the passage line briefly. This is apart from TVATCRB-3 in which fish spent time in the near wake on a similar level as TVATCRF-3 but did not swim significantly shorter distances compared to C-3. The proportion of the area explored was highest with a stationary turbine downstream of the turbine and may be explained similarly by the fish finding hydrodynamic benefit generated by the turbine wake, which is absent when the turbine is stationary^22^. Finding favourable hydrodynamic conditions may then lead to a reduced need to explore areas with higher flow velocity. This can be supported by lower TBF for treatments with paired turbines that produce a larger wake region and from previous studies where rainbow trout entrained behind moving foils and cylinders^56,57,66^.

The current findings also agree with existing literature^29–31,33,37,41^ regarding 100% survival and no visible injuries after the trials. Although the turbine used in our study was a scaled version of a VAT and is therefore not representative of a full-scale turbine in terms of injury and mortality, the findings remain encouraging and support findings from a field study with similar rpm ranges^40^.

The most widely observed response to turbines is avoidance^29,31,32^, and this was also seen in our single fish treatment (SVAT-1), where the distance to the turbine was significantly higher and turbine interactions significantly lower than control (three fish with a stationary turbine). When shoaling, however, the trout had increased turbine interactions, spent more time closer to the turbines, and made wider use of the near wake and bow wake, particularly for co-rotating turbines. Despite a higher count of interactions with turbines for all shoaling treatments, only fish in TVATCO-3 and TVATCRF-3 spent more time in the area very close to the turbines, suggesting either that the flow within and between the turbines was more turbulent in other treatments, or that the turbine was still perceived as dangerous by the fish and that it may not have been visible to them until very close^37^. Another explanation is that more time spent in the bow wake led to fish crossing the boundary with the turbine region leading to a higher apparent amount of time spent in the turbine area. The single fish in SVAT-1 spent more time resting than all shoaling treatments, a behaviour also noted in previous studies^31,37^ but which is not entirely explained by the hydrodynamics as the shoals did not seem to create a hydrodynamic advantage in terms of TBF. This suggests that increased resting time may be socially driven by the absence of shoal mates as revealed by the analysis of the resting time compared to the proportion of time spent shoaling.

Fish did not make use of the far wake of the rotating turbines. The velocity in the wake of a single VAT is 95% recovered by 5D (five turbine diameters) downstream of the turbine^21^ and with the TVATCRF turbine configuration, momentum recovery occurred by 5D^27^. This indicates that the potential benefits of a turbine wake, like lower velocities, have diminished by the downstream end of the working area, especially for TVATCRF-3. In the upstream end of the far wake region, however, there were reduced TBF values for treatments with twin turbines, showing that velocity reductions could still be exploited in what Müller et al. (2021) termed the transition zone.

Time spent in the bow wake was very consistent across treatments except for TVATCO-3 in which significantly more time was spent there. With the TVATCO-3 setup, there is a velocity reduction in the induction region upstream of the two turbines, potentially providing a more stable swimming environment for the fish than the TVATCRF, TVATCRB, and SVAT layouts^26^. The SVAT setup has reduced velocities upstream of the turbine but to a lesser magnitude and spatial extent than twin turbines, TVATCRF has a stronger velocity reduction but the velocity gradient is larger and the reduction more localised whereas TVATCRB has a lower velocity reduction and flow accelerating between the two turbines driven by the turbine rotation^26^, potentially explaining why bow waking was more common with the TVATCO setup. The TBF in the bow wake was only reduced while the trout were shoaling, potentially indicating that it is still an unstable area to swim in for single fish whose TBF was not overall lowered in the bow wake. A probability density function analysis of the midline fish body data did not, however, reveal any obvious destabilisations of the fish swimming in the bow wake. Some shoaling studies suggest that one mechanism of information transfer and hydrodynamic benefit of shoaling is the reduced pressure difference and lower velocity directly in front of fish behind the leader^58^. These hydrodynamic conditions are partially replicated by the turbine’s upstream induction region, so there is a possibility that fish were in effect ‘shoaling’ with the turbines as these had diameters in the same order of magnitude as the fish (the ratio of mean fish size to turbine diameter is 0.56) and turbine rotors were spaced relatively close to each other. Similarly, time spent in the near wake was higher for all treatments with paired turbines as they produced a larger velocity reduction in the near wake compared to a single turbine^27^. Tailbeat frequency data is lower with more time spent in the near wake with TBF in the near wake being significantly lower in treatments with paired turbines. This is most likely due to the reduced velocities but could also be due to the vortices shed by the turbine blades which match the TBF of the fish (59 rpm with three blades leads to 2.95 Hz and the TBF was around 3.4 Hz) and fish have been shown to use vortex streets to reduce energy consumption^57,67^. The vortices behind the turbines, however, are likely unsuitably placed for fish to draw a hydrodynamic gain from them so velocity reduction is the most likely explanation for the lower TBF values.

Overall, TBF was not affected by shoaling alone. Most reductions in TBF were associated with the turbine wake region indicating that shoaling in this study did not bring direct energetic advantages to the fish. This is contrary to previous findings reporting shoaling reducing swimming effort^51,53,54,59^ but in line with a previous study where no metabolic benefit was found in rainbow trout shoaling^52^. An analysis of TBF by treatment filtered by shoal size also did not find differences. It is possible that a shoal of three fish was not of sufficient size to bring energetic benefits but there is evidence to show that it did affect behaviour by increasing turbine interactions and decreasing resting time. Hence, shoaling does not have an exclusively hydrodynamic role but that there are social benefits to being close to other fish.

The longer a fish shoaled, the more equally distributed the role of leader was among the shoalmates. This offers insight into the shoaling dynamics of the trout, in very short durations of shoaling there was insufficient time for fish to take their turn as leader but for longer durations, the fish were able to share this role equally, independent of treatment or location within the working area. These results also highlight the difference between the behaviour of a shoal and an individual fish. Shoaling is often overlooked in flume studies assessing fish movement and behaviour but this study has shown marked differences in behaviour when shoaling thus studies with a single fish only reveal part of the picture. Shoaling is common in many species of fresh and saltwater fish and should therefore be considered more widely in future studies of fish behaviour at turbines.

The ecological considerations arising from this study are overall positive. Passage remains unaffected with one or two laboratory scale turbines in a relatively wide channel and the probability of strike are very low. There is also evidence to suggest that turbine wakes may reduce energy expenditure as fish were more likely to hold station in these areas but this may potentially lead to increased predation hotspots^36^. With passage and survival rates far exceeding those of most hydropower plants, hydrokinetic turbines could score better in environmental evaluations^6^, which can be used as management tools by regulators in appraising different schemes. It must be noted, however, that this laboratory study cannot replicate the real operation of a full scale turbine which would produce a comparatively more energetic wake and operate in a more turbulent environment. Another limitation of the study is that the relative swimming performance of the fish used in this study is too high.

## Conclusion

An experimental study of single and twin hydrokinetic turbines show that these devices neither limit upstream or downstream passage of rainbow trout. Paired turbines led to increased engagement by shoals of trout that used the bow- and near-wake low velocity regions to potentially reduce energy consumption over a single turbine because of the larger extension and lower velocities in the wake regions. Areas of low velocity where generally preferred by the fish. Tailbeat frequency was reduced by low velocity areas in the turbine’s downstream wakes while remaining unaffected by shoaling alone. Single fish swimming alone displayed increased resting times and fewer approaches to the turbine whilst shoals were bolder and more likely to exploit the potential hydrodynamic benefits of the turbine wakes. Our findings have the potential to inform environmental impact assessments involving hydrokinetic turbines being a suitable technological solution to generate energy from rivers and tidal-streams while reducing disruptions to fish populations in the aquatic environment.

## Methods

### Fish origin and maintenance

Fish trials were performed between 29th March and 7th April 2021 from 8:00 to 17:00 for individual fish and between 22nd March and 30th April 2021 from 8:00 to 17:00 for shoaling fish. The fish used in these trials were female triploid Rainbow trout (*Oncorhynchus mykiss*, *N* = 80) purchased from Bibury Trout Farm, UK. Trout total length was 67.5 ± 10.7 mm. Before the experiment, they were housed in a Recirculating Aquaculture System (RAS) in tanks of 60–80 L at 40 fish per tank in Cardiff School of Biosciences on a 12 h:12 h light: dark cycle at 15 ± 1 °C in water being constantly filtered by the RAS. They were fed daily with trout pellets in the morning. Shortly before the start of the flume trials, the trout were transported to a holding tank in Cardiff School of Engineering where they were separated into size-matched shoals, isolated in plastic mesh cylinders within a larger holding tank of 500 L. The trout were maintained in dechlorinated water (Tetra AquaSafe Seachem Prime Concentrated Conditioner), aerated with multiple air pumps, cooled to 13 ± 1 °C by a D-D Aquarium Solution DC 750 chiller, and filtered by an external canister filter (Aquamanta EXF 600). Water quality was checked weekly with a water quality test kit (Nutrafin). The trout were kept on a 14 h:10 h light: dark cycle and fed trout pellets every morning. The tank was provided with plastic tubes and other refugia. The shoals were acclimated for two weeks in this setup before any testing occurred to allow fish to familiarise with their shoal. The shoaling trials were carried out in three batches over the course of two months. After the end of the trials, the fish were transferred back to the RAS at Cardiff School of Biosciences. In the case of the single fish treatment, the 20 trout were housed in the 500 L tank with no plastic mesh sub-dividers but otherwise kept in the same way as the shoals. When the single trout had completed the experiment, they were transferred directly back to the School of Biosciences.

### Experimental setup

**Fig. 5:**
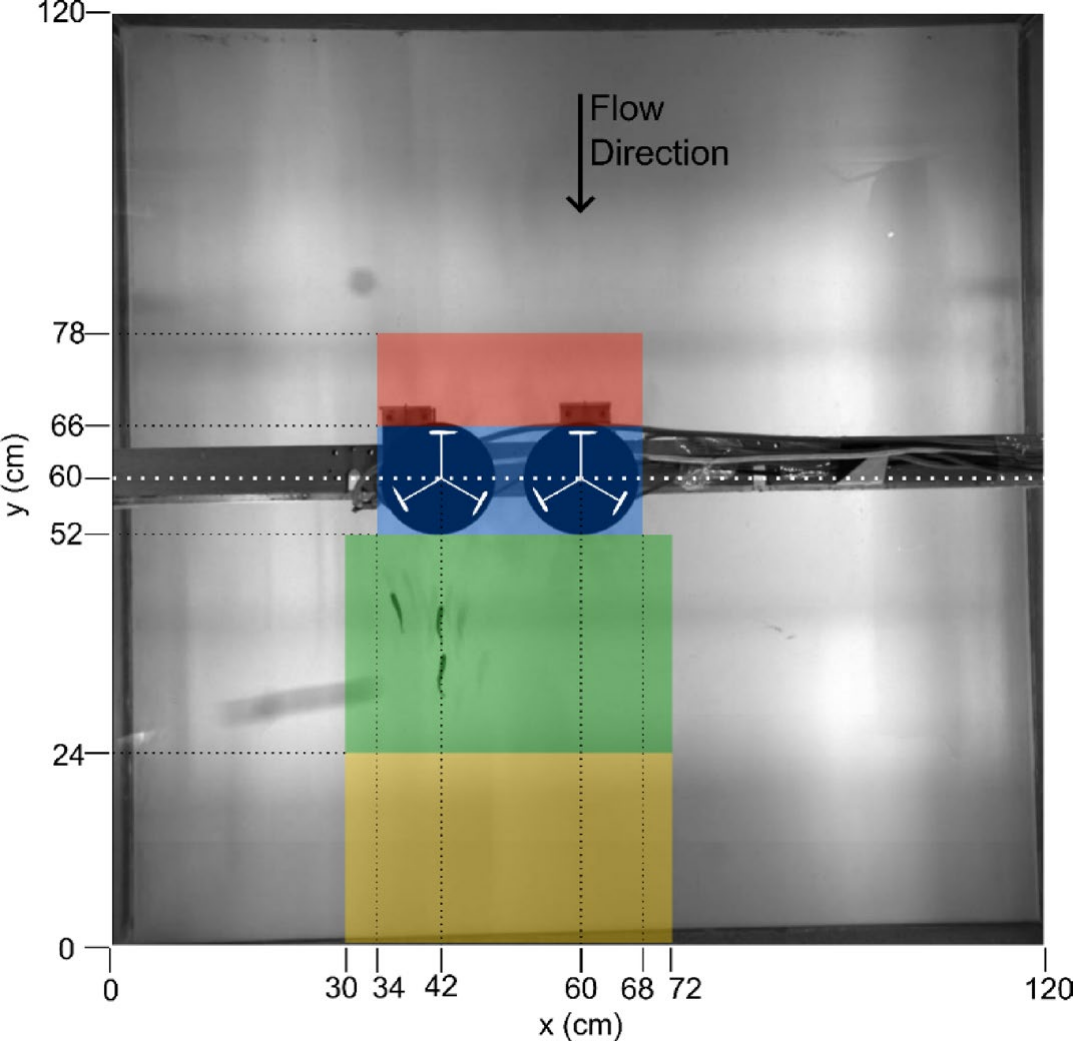
Schematic of the working area with all measurements in cm and the water flowing from top to bottom with the lateral direction defined as the x axis and the streamwise direction as the y axis. The two turbines are both depicted as rotating counter-clockwise (TVATCO-3 treatment, see Table 1). The single turbine tests were conducted with a turbine positioned on the flume centreline and the turbine centred at (y = 42 cm, x = 60 cm) was removed. The coloured areas represent the areas defined in Table 2, where yellow = far wake, green = near wake, blue = turbine, and red = bow wake.

A recirculating open channel flume (10 m long, 1.2 m wide, and 0.3 m high with a 1/1000 bed slope) at Cardiff School of Engineering was used in this study. The working section of the flume was bounded up and downstream by honeycomb flow straighteners and on the flume walls by glass. The flume bed in the working section was a white PVC sheet and the water surface was covered by a transparent acrylic sheet. The upstream end of the working section was located 4.4 m from the upstream end of the flume and measured 1.2 m long and 1.2 m wide with a flow depth of 0.23 m. Laboratory scale Vertical Axis Turbines (VATs) were constructed with a diameter and height of 0.12 m, with three blades of 0.03 m chord length and a NACA0015 profile. The turbine diameter was scaled geometrically to fish length. Full details of scaling are given in Supplementary materials 1. With a single VAT, it was positioned at the centre of the working area, at (x = 60 cm, y = 60 cm) in Fig. 5, whereas when a second turbine was present it was inserted at (x = 42 cm, y = 60 cm) in Fig. 5, a spacing of 1.5 turbine diameters. Each blade was mounted to a central shaft by two 3 mm diameter struts and was laser sintered from white PA2200. The central shaft was mounted in a bearing embedded in the flume bed and held the bottom of the blades 20 mm from the bed. The upper end of the shaft was connected to an encoder and a DC motor (Kübler, 5-30VDC, 100 mA and Nider DMN37K50G18A, DC 12 V respectively) which was held by a beam resting on the sides of the flume. The turbine operated at a fixed rotational speed of 59 rpm, combined with a bulk velocity of 0.19 ms^− 1^ (Q = 53 Ls^− 1^), this gave the optimum tip speed ratio of 1.9^21,27^. The working area was illuminated from both sides by a total of four spotlights (Neewer Bi-Colour LED) and recorded at 55 frames per second (fps) from an overhead camera (Baumer VLXT-50 M.I). The water in the flume was dechlorinated using Prime Dechlorinator and cooled to 14 ± 1 °C by a D-D Aquarium Solution, DC2200.

### Experimental procedure

For shoaling treatments, *n* = 19 groups of three fish and for the single fish treatment *n* = 16 individual fish were included in the analysis. Each shoal (or single fish) was transferred from the holding tank to the flume in a bucket of water and released at the downstream end of the experimental area. The fish were then allowed to acclimate for a total of 20 min - the first 5 min at a reduced flowrate of 24.5 Ls^− 1^ and corresponding turbine rotation of 7 rpm, the following 5 min at 37 Ls^− 1^ and 30 rpm, and the final 10 min at the nominal flowrate of 53 ls^− 1^ and 59 rpm. At the end of the acclimation period, the fish were removed from the working area into a bucket whilst the perspex sheet was placed above the area. The recording was started with a 10 min 30 s timer, with the first 30 s used to place the trout at the downstream end of the experimental area and to start the experiment (these initial 30 s were discarded from the analysis). After the 10-minute experiment, the trout were removed from the flume and transferred back to their holding tank. In the case of single fish, the fish length and weight were measured with a calliper and a scale and then immediately transferred to a tank. The single fish only took part in one treatment, namely a single fish with a single turbine (SVAT-1, Table 1), with more information on the single fish treatment (SVAT-1) featured in a previous study^24^ where the results of this treatment were used to investigate the impact of flume width on fish swimming behaviour. In contrast, the shoals of three fish took part in all treatments (Table 1), with each shoal only taking part in one treatment per day, completing all five different treatments over five consecutive days. The treatment order was randomised for the shoals to remove any temporal, learned behaviour, or tiredness bias.

**Table 1:** Details of the treatments investigated in the study, where in the treatment names C = Control, SVAT = Single VAT, TVAT = Twin VAT, CO = Co-Rotating, CRB = Counter-Rotating Backwards, CRF = Counter-Rotating Forwards, and the number denotes the number of fish. In the rotation direction column, where two turbines are present, their relative rotational directions are described from left to right looking upstream (Fig. 5). For shoaling treatments *n* = 19 shoals and for the single fish treatment *n* = 16.

Treatment	No of Fish	No of Turbines	Rotation direction	Turbine Rotational speed (rpm)	Flowrate (Ls^− 1^)	Bulk Velocity (ms^− 1^)	Flow Depth (m)
C-3	3	1	Stationary	0	53	0.19	0.23
SVAT-1	1	1	Counter-Clockwise	59	53	0.19	0.23
SVAT-3	3	1	Counter-Clockwise	59	53	0.19	0.23
TVATCO-3	3	2	Counter-Clockwise	59	53	0.19	0.23
TVATCRB-3	3	2	Clockwise and Counter-Clockwise Backwards	59	53	0.19	0.23
TVATCRF-3	3	2	Counter-Clockwise Forwards and Clockwise	59	53	0.19	0.23

### Data analysis

The video recordings of the fish were processed removing the initial 30 s using Matlab R2024a^68^, and then they were then analysed in TRex^69^ with which the positions and midline points of all fish for each frame were inspected, corrected where necessary, and exported as Matlab files. The data for each fish were then analysed in Matlab to obtain the parameters shown in Table 2, some of which are based on a grid defined with the origin at the left corner of the downstream end of the working area, also shown in Fig. 5.

**Table 2:** The variables calculated from the tracking and posture data of rainbow trout in the current study. The *x* and *y* values (given in cm) in the description are in reference to the coordinates shown in Fig. 5. Areas are also defined in Fig. 5.

Variable	Description	Diagram
Distance (m)	Total distance travelled by each fish, calculated by summing the distance between each frame over the 10 min of the trial.	
Proportion explored (-)	The 14,400 cm^2^ working area was split into 100 cm^2^ squares and the number of squares entered by each fish counted and expressed as a proportion of the total number of squares.	
Cross count (-)	Number of times each fish passed upstream or downstream of the turbines (*y* = 60 cm).	
Turbine count (-)	Number of times each fish entered the turbine rotor region, defined as (52 < *x* > 68 cm, 52 < *y* > 66 cm) when one turbine was present and as (34 < *x* > 68 cm, 52 < *y* > 66 cm) for two turbine configurations.	
Turbine time (s)	Time spent in the turbine area (defined above).	
Near wake time (s)	Time spent by each fish in the near wake (30 < *x* > 72 cm, 24 < *y* > 52 cm).	
Far wake time (s)	Time spent by each fish in the far wake (30 < *x* > 72 cm, 0 < *y* > 24 cm).	
Bow wake time (s)	Time spent by each fish in the bow wake (directly in front of the turbine) (34 < *x* > 68 cm, 66 < *y* > 78 cm).	
Time upstream (s)	Time spent by each fish upstream of the turbine (60 < *y* > 120 cm).	
Time downstream (s)	Time spent by each fish downstream of the turbine (0 < *y* > 60 cm).	
Time in 3 fish (-)	The amount of time for which all three fish were within three fish lengths of each other (based on the average total fish length of that shoal) expressed as a proportion of the total time. This analysis was also repeated for each of the areas defined above by only considering time in that area.	
Time in 2 fish (-)	The amount of time for which two fish were within three fish lengths of each another (based on the average total fish length of that shoal) expressed as a proportion of the total time. This analysis was also repeated for each of the areas defined above by only considering time in that area.	
Time alone (-)	The amount of time for which all fish were more than three fish lengths away from one another (based on the average total fish length of that shoal) expressed as a proportion of the total time. This analysis was also repeated for each of the areas defined above by only considering time in that area.	
Time resting (s)	Number of frames during which the swimming speed and tailbeat frequency of the fish was approximately zero.	
Dominance (-)	The proportion of time during shoaling for which each fish was upstream of its shoalmates was calculated for each fish and the second highest proportion was subtracted from the highest proportion.	
Tailbeat frequency (TBF) (Hz)	The tail movement was extracted from the posture tracking and a fast Fourier transform performed on the signal. The dominant frequency was extracted for every second of data. This method was validated against human analysed swimming clips. This analysis was also repeated for each of the areas defined above by only considering frames in that area.	

### Statistical analysis

The statistical analysis for this study was performed in RStudio (R Version 4.2.2)^70^. The data was imported into R and checked for normality. Due to the repeated use of the same shoals, Generalised Linear Mixed Models (GLMM) were utilised to account for pseudo-replication by including shoal ID as a random effect. To perform the GLMMs the lme4, nlme, and lmertest packages were used^71–73^. The study was performed in batches, so batch effects were first inspected to verify that there were no statistical differences between batches for all response variables (GLMM, *p* > 0.44). The data was normally distributed so a Gaussian GLMM produced the best residual distributions compared to other GLMMs tested. Count data was analysed using a Poisson GLMM which outperformed negative binomial and other GLMMs. To further analyse TBF, Matlab was used to perform a Probability Density Function (PDF) for TBF in different areas of the working section. In all cases the p value use to determine significance was 0.05, where multiple relationships are reported on (for instance the relationship of all treatments compared to control), the upper bound of the p value for significant results is given.

### Hydrodynamics

**Fig. 6:**
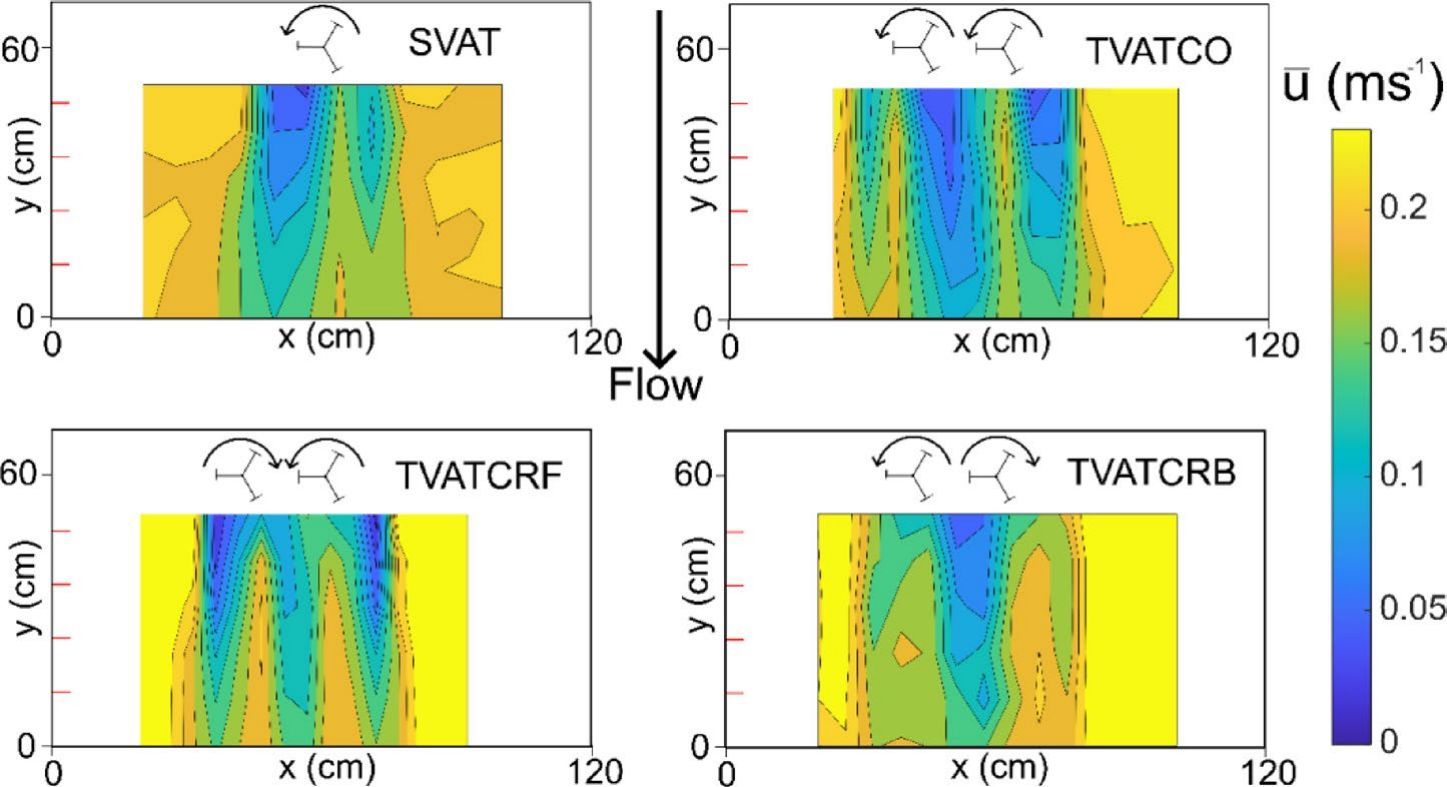
Contour plots of time-averaged streamwise velocity taken at mid turbine depth for all turbine configurations. The coordinate system on each plot aligns with that of Fig. 5. Red marks on the y axis represent diameters downstream of the turbines. Acoustic Doppler Velocimetry was used to collect this data by Müller et al. (2021). The turbine spacing was 1.5 turbine diameters and longitudinal extent of the wake is shown downstream of the turbines up to the flow straightener at y = 0 cm (5 turbine diameters, Fig. 5).

A previous experimental study^21^ fully characterised the three dimensional wake shape from Acoustic Doppler Velocimetry data for the turbine configurations tested. Data was available for the following distances downstream of the turbines: 1D, 1.5D, 2D, 3D, 4D, and 5D where D is the turbine diameter equal to 12 cm. Figure 6 provides contours of time-averaged streamwise velocity for flow depth z = 100 mm. The slowest velocities in the wake are generated in the downstream region corresponding to the upstream motion of the blades. The TVATCRB setup generated the wake with the smallest horizontal footprint while the velocity deficit is largest for the TVATCO^27^. The highest turbulence intensity across the wake is from the TVATCRB^27^.

### Animal ethics statement

All work was performed under the relevant guidelines and regulations and approved by Cardiff University Animal Ethics Committee and linked to UK Home Office PP816714 and followed ARRIVE guidelines.

## Supplementary Information

Below is the link to the electronic supplementary material.

Supplementary Material 1

## Data Availability

Data from this study is freely available at DOI: 10.17632/xmrptb6mjw.1.
