# Supplementary material for: Impact of hydrokinetic turbines on rainbow trout behaviour
Source: Sci Rep. 2026 Mar 16;16:13652. doi: 10.1038/s41598-026-43568-8 (PMC13125489; doi:10.1038/s41598-026-43568-8)
Supplement: Supplementary file 1 — Supplementary Material 1 [file 41598_2026_43568_MOESM1_ESM.pdf]

Impact of hydrokinetic turbines on rainbow trout behaviour - Supplementary Materials 1.

Scaling methods and calculations

Turbine and fish geometric scaling: The prototype turbine at the site of interest in Columbia at the San Juan River in Vereda el Salado was estimated to 0.67m diameter based on the hydraulic conditions at this specific site (although more widely, turbines of this type range from 0.36-4.4 m diameter). Turbine size was restricted by the dry season which showed a typical mean flow depth of 3.6 m and mean velocity of 1.47 m/s. By using a geometric scaling factor of 0.18, this resulted in a model turbine diameter of 0.12m suitable for our flume. Applying this scaling factor to the model fish used (0.067m length) would correspond to a 0.37 m long adult salmonid. Typical upstream migrating salmonids show a range of 0.4-1.5 m and hence the model fish are at the lower end of this spectrum for the prototype turbine but scale well for similar ranges of fish and turbine sizes. Applying the same geometric scaling factor to both fish and turbine allows to preserve a fish-turbine-ratio of 11:20.

Froudian flow scaling: Flow was scaled from the San Juan River site based on winter conditions (Cross sectional area = 1753.4 m^2, mean flow depth = 9.43 m, mean velocity = 1.62 m/s, flow rate = 2843.9 m^3/s, wetted perimeter = 192.5 m, hydraulic radius = 9.11 m) which gives a Froude number of 0.17. Laboratory flow conditions were at a flow velocity of 0.19 m/s and flow depth of 0.23 m (in a flume 1.2 m wide) to give a Froude number 0.15, similar to the San Juan River site. The exact Froude number could not be achieved due to the limitations of obtaining uniform flow conditions in the laboratory flume while maintaining the correct turbine submersion.

Turbine rotation scaling: The rotational velocity of the turbine was determined based on maintaining the optimal Tip Speed Ratio for the turbine of 1.9, so the turbine was set to rotate at 59 rpm.

Confidentiality: C2 - Internal
